# 3D Bioprinting of Novel Biocompatible Scaffolds for Endothelial Cell Repair

**DOI:** 10.3390/polym11121924

**Published:** 2019-11-22

**Authors:** Yan Wu, Lamia Heikal, Gordon Ferns, Pietro Ghezzi, Ali Nokhodchi, Mohammed Maniruzzaman

**Affiliations:** 1 Department of Pharmacy (Chemistry), School of Life Sciences, University of Sussex, Brighton BN1 9QJ, UK; yw354@sussex.ac.uk; 2 Brighton and Sussex Medical School, Brighton BN1 9RH, UK or G.Ferns@bsms.ac.uk (G.F.); p.ghezzi@bsms.ac.uk (P.G.); 3 Faculty of Pharmacy, Department of Pharmaceutics, Alexandria University, El Sultan Hussein St AZARITA-Qesm Al Attarin, Alexandria Governorate 21521, Egypt; 4 Drug Applied Research Center and Faculty of Pharmacy, Tabriz University of Medical Sciences, Tabriz 51664, Iran; 5 Pharmaceutical Engineering and 3D Printing (PharmE3D) Lab, Division of Molecular Pharmaceutics and Drug Delivery, College of Pharmacy, The University of Texas, Austin, TX 78712, USA

**Keywords:** 3D bioprinting, biocompatible, endothelial cell, DMOG, EPO, scaffolds, polylactic acid

## Abstract

The aim of this study was to develop and evaluate an optimized 3D bioprinting technology in order to fabricate novel scaffolds for the application of endothelial cell repair. Various biocompatible and biodegradable macroporous scaffolds (D = 10 mm) with interconnected pores (D = ~500 µm) were fabricated using a commercially available 3D bioprinter (r3bEL mini, SE3D, USA). The resolution of the printing layers was set at ~100 µm for all scaffolds. Various compositions of polylactic acid (PLA), polyethylene glycol (PEG) and pluronic F127 (F127) formulations were prepared and optimized to develop semi-solid viscous bioinks. Either dimethyloxalylglycine (DMOG) or erythroprotein (EPO) was used as a model drug and loaded in the viscous biocompatible ink formulations with a final concentration of 30% (*w*/*w*). The surface analysis of the bioinks via a spectroscopic analysis revealed a homogenous distribution of the forming materials throughout the surface, whereas SEM imaging of the scaffolds showed a smooth surface with homogenous macro-porous texture and precise pore size. The rheological and mechanical analyses showed optimum rheological and mechanical properties of each scaffold. As the drug, DMOG, is a HIF-1 inducer, its release from the scaffolds into PBS solution was measured indirectly using a bioassay for HIF-1α. This showed that the release of DMOG was sustained over 48 h. The release of DMOG was enough to cause a significant increase in HIF-1α levels in the bioassay, and when incubated with rat aortic endothelial cells (RAECs) for 2 h resulted in transcriptional activation of a HIF-1α target gene (VEGF). The optimum time for the increased expression of VEGF gene was approximately 30 min and was a 3-4-fold increase above baseline. This study provides a proof of concept, that a novel bioprinting platform can be exploited to develop biodegradable composite scaffolds for potential clinical applications in endothelial cell repair in cardiovascular disease (CVD), or in other conditions in which endothelial damage occurs.

## 1. Introduction

Three-dimensional bioprinting (3DP), which has emerged as an innovative additive manufacturing technology [1,2,3], is revolutionizing the field of tissue engineering and thus the future of medicine and medical implants. Similarly, novel biocompatible bio-inks (with or without a drug) is also equally transforming tissue engineering applications and can be used to fabricate complex geometries of personalized medical devices, e.g., scaffolds, providing novel platforms beyond the current state of the art. There is an opportunity for technological innovation in the fabrication of novel scaffolds or biomaterials using 3DP that requires a convergence of expertise in biomaterial, pharmaceutical, and vascular biological fields.

A major challenge for tissue engineering has been to mimic the micro and macro environment of human tissues via a widely used method to generate cell-seeded scaffolds both in anatomically complex geometries and intra-cellular architectures with controlled cell distribution. Studies have revealed that the critical characteristic of a biomaterial, as well as the control of the inner micro- and macro-scale features of the engineered-tissue, is considered a key quality parameter to fabricate complex anatomical, patient-specific structures with high shape fidelity in tissue engineering applications [4,5]. In response to this currently unmet need, advances in additive manufacturing and thus 3DP have inspired scientists to employ this innovative technology for biomaterial and tissue engineering strategies [6,7]. Bioprinting, in particular, has gained attention for its ability to control and deposit sequential layers of biomaterials, allowing the tailoring of a specific geometry to an object and permitting the placement of cells and biological molecules [8,9]. As a result, 3DP offers numerous possibilities for the future of tissue engineering and organ regeneration. Bioprinting can be combined with Computer-Aided Design (CAD) technology using patients’ medical images to allow the biofabrication of biomimetic-shaped 3D structures unique to the target tissue or organ in a personalized manner. Because of the challenges encountered when bioprinting, considerable improvements need to be made in order to bioprint complex constructs or cell-laden 3D tissue constructs by means of developing suitable biomaterials and bioink formulations with optimum properties such as viscosity for successful 3D (bio)printing [10,11]. 3D bioprinted scaffolds built for individual patients are favoured over customization of mass-produced products when meeting the specific needs of each patient. The benefits of its clinical application include easy adaptation and fixation, reduced surgical time, and favorable aesthetic results. 

Coronary heart disease (CHD) is initiated when the cell lining of arteries (the endothelium) is injured. Endothelial regrowth appears to be an important process limiting CHD. When cells are injured, they activate an inflammatory reaction which induces the expression of the innate repair receptor (IRR), which activates tissue protection and repair [12]. Despite the early and strong expression of IRR within injured tissues, local production of tissue protective cytokines (TPCs) such as erythropoietin (EPO) is delayed, transient and relatively weak [13]. This provides an opportunity to intervene with exogenous TPCs that act as innate repair activators, targeting the fundamental processes of tissue injury at a level that controls both the self-damaging and the regenerative components, representing a promising therapeutic approach [14]. EPO has been shown to be tissue-protective in models of ischaemic, traumatic and inflammatory injury [15]. Hypoxia enhances the reparative response of ECs to EPO [16,17]. This is likely to be mediated by hypoxia inducible factor (HIF)-1. Dimethyloxalylglycine (DMOG) is a HIF-1α inducer and mimics conditions similar to hypoxia [16,17,18,19,20].

The use of biomaterials such as biocompatible or biodegradable copolymer (e.g., polylactic acid, pluronic F127 [21]), is a common strategy for reducing the risk of thrombosis and restenosis. Blood compatibility remains a major issue and several surface modifications have been used to mitigate this problem. We aimed to use a novel bio-therapeutic material, with intrinsic tissue protective activity, to fabricate a novel scaffold by means of an optimized 3D bio-printing technology that can promote EC repair and may offer a promising alternative therapy to existing biomaterials such as polylactic acid (PLA) and pluronic. Among other biomaterials, both polylactic acid (PLA) and pluronic (F127) biopolymers have been used as a suitable polymeric carrier for the development of bioinks because of its superior biocompatibility and printing fidelity [21]. We also wished to apply this 3DP technology to prevent restenosis, based on triggering the endogenous repair mechanisms of the endothelial cells of the artery wall.

## 2. Materials and Methods

### 2.1. Materials 

Polylactic acid (PLA) MW 60,000, polyethylene glycol (PEG) MW 400 were purchased from Sigma Aldrich (Gillingham, UK). Pluronic F127 (F127)-based biomaterials were purchased from SE3D (Santa Clara, CA, USA). Dimethyloxalylglycine (DMOG) was purchased from Sigma Aldrich (Dorset, UK) and Erythropoietin (EPO) was purchased from Araim Pharmaceuticals (New York, NY, USA). All materials required for cell culture assessment and analytical studies were purchased from Sigma Aldrich (Dorset, UK) unless otherwise stated. All solvent and chemical were of analytical grade and used as received. 

### 2.2. Preparation of Bioinks Containing EPO and DMOG

The biomaterial matrix was prepared from a mixture of poly (lactic acid) (PLA) and Polyethylene glycol 400 (PEG) and Pluronic F127 (F127) in different concentrations as shown in Table 1. Briefly, PLA was prepared as a 15% solution by dissolving the PLA pellets in chloroform. PEG was then dissolved in the PLA solution. PLA: PEG matrix was prepared in the following concentrations: 7:0, 6:1 and 5:2 *w*/*w* ratios. A wide range of inks was developed and only the best four (for PLA/PEG only 5:2 *w*/*w* ratio was selected) were used for this part of the study (Table 1). Model drugs, i.e., EPO and/or DMOG, were loaded on the biomaterial matrix (bioink) to reach a final concentration of 20%–30% (*w*/*w*). 

### 2.3. Rheology Measurement of the Inks 

Once optimized, the ink formulations were subject to a rheology measurement study. The viscosity of the inks was measured both at constant and increasing shear rates using a plate rheometer (RheoStress^®^ RS 1, Karlsruhe, Germany) with a plate–plate distance of 0.052 mm. The viscosity of the pastes was determined by applying a constant shear rate of 10 s^−1^ for 500 s. After an initial amplitude sweep test to detect the viscoelastic region, oscillatory frequency sweep tests (*f* = 0.01–10 s^−1^; 1.0 Hz) were performed at 25 °C on all ink formulations as shown in Table 1. 

### 2.4. Extrudability/Injectability of the Inks Developed for Bioprinting 

Injectability of the bioink formulations is an important factor to consider in order to ensure the required dose is delivered effectively and more precisely, and with ease. In general terms, the force which is applied to a syringe plunger during the injection of a formulation via a needle is classified in three ways: stiction, overcoming the resistance force of the syringe plunger; plateau force, energy that accumulates as the formulation glides through the needle under a constant force and finally end constraint force. These three types of forces were recorded manually and were classified as ‘pass’ or ‘fail’, based on whether they did or didn′t expel the formulation steadily out of the syringe, respectively. Based on the preliminary observation and the analysis, only those 4 formulations (Table 1) were used for 3D bioprinting. 

### 2.5. 3D Bioprinting of Scaffolds

**Figure 1:**
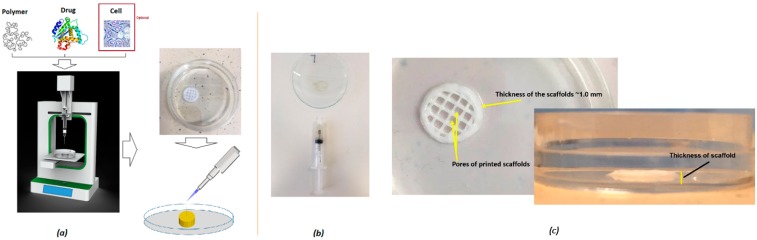
Schematic diagram of (**a**) the optimized 3D bioprinting process, (**b**) the extrudable bioink, and (**c**) the printed scaffold with 3D texture.

An optimized 3D bioprinting platform (Figure 1) was used to fabricate all macro-porous scaffolds with a diameter of 10 mm. The thickness of all developed scaffolds was set at ~1–3 mm as a default in order to avoid any possible effect of the varying thickness of the scaffolds on the actual release of the drug. The adopted printing process was later applied for the printing of different scaffolds with various geometries and intricacies of the constructs. The print resolution was set at 100 µm and the construct was directly printed (via r3bEL mini bioprinter, SE3D, Santa Clara, CA, USA) in a petri dish placed on the print bed in ambient temperature (23 ± 1 °C). The various developed viscous printing inks were drawn into a 22-gauge printing syringe with an internal diameter of ~640 µm from which the inks were extruded at a speed of ~100 mm/min to print the constructs. Computer-Aided Design (CAD) was utilized to develop the design of the scaffolds with the required geometry (rectangular pores). Once the fabrication process was optimized, all printed scaffolds were immediately removed from the print bed and the petri dish was stored at 37 °C in an incubator for 24 h to cure the 3D printed scaffolds prior to utilizing it for further analysis. 

### 2.6. Surface Morphology of the Inks and Scaffolds

The surface morphology of the fabricated scaffolds was studied using a scanning electron microscope (Jeol JMS 820, Freising, Germany). The samples were placed on a double-sided carbon tape and sputter-coated with gold using a sputter coater (Edwards S-150 sputter coater, Edwards High Vacuum Co. International, Sanborn, NY, USA). After the samples were sputter-coated, they were placed into the SEM where the surface structure was then observed and recorded at various magnifications using the SEM operating at 3 kV. An optical microscope (JEOL JEM1400-Plus, 120 kV, LaB6, Peabody, MA, USA) was also utilized to investigate the surface of the viscous inks as well as scaffolds to visualize the texture of the ink formulations prior to the 3D printing and the surface of the developed scaffolds to determine the distribution of the deposited substances on the surface of the scaffolds.

### 2.7. Mechanical Properties of the Scaffolds Developed

Uniaxial compressive tests were applied to scaffolds and Young′s modulus and compressive strength were obtained from the data. For the purpose of this study, the scaffold strips (3–5 mm in length) were attached to a 75-mm-diameter adhesive rig probe with a double-sided adhesive tape on a TA.HD.plus Texture Analyser (Stable Micro Systems, Surrey, UK) fitted with a 5-kg load cell in compression mode. The probe, lined with the scaffolds, was lowered towards the surface at a pretest speed of 0.5 mm/s, test speed of 0.5 mm/s and post speed of 1.00 mm/s. The maximum force required to penetrate the scaffolds was determined. The mechanical analysis was conducted at room temperature (23 ± 1 °C) and run in triplicate (n = 3).

### 2.8. Thermal Analysis

Solid state of the drugs was analyzed via differential scanning calorimetry (DSC) (DCS 4000, Perkin Elmer, Waltham, MA, USA). The study was performed on the chosen bioink formulations, which were utilized for the development of the scaffolds. The crystallinity of the substances used in the formulations was examined using data presented in each of the DSC traces. Samples weighing between 3 and 6 mg were sealed in an aluminium pan and placed in the DSC machine with a scanning rate of 10 °C/min (from 25 to 265 °C) under nitrogen atmosphere. 

### 2.9. Drug Release Study from the Scaffold

The scaffolds were placed into PBS (500 µL). The PBS containing the released drug was removed at different time intervals (5, 15, 30 min, 1, 2, 4, 24 and 48 h) and replaced with fresh PBS each time. The aliquots removed were kept at −20 °C until further analysis. Rat aortic endothelial cells (RAECs) were seeded into 24-well plates and cultured until approximately 80% confluency in 21% O_2_. 100 µL of the aliquots with the released drug at different time intervals was added on RAECs and left for 2 h. As DMOG is a HIF-1α inducer, its release from the scaffold into PBS solution was quantitatively measured indirectly using a bioassay for measuring HIF-1α. In another set of experiments, DMOG was measured indirectly by measuring the gene expression of VEGF using real-time qPCR. EPO release from the scaffold in PBS solution was also quantitatively measured directly using an ELISA kit for EPO.

### 2.10. Real-Time qPCR

Treated cells were lysed using TRIzol (Invitrogen/ Life Technologies, Dartford, UK) and RNA was extracted and purified as described previously [22]. RNA quality and concentration were determined using a NanoDrop ND-1000 (NanoDrop Technologies). Reverse transcription and real-time quantitative PCR (qPCR) for VEGF and β2-microglobulin (a housekeeping gene), were carried out on RNA samples using Taqman gene expression assays (Applied Biosystems/Life Technologies, Dartford, UK) as previously reported [23]. For gene expression quantification, the comparative threshold cycle (ΔΔCt) method was used following Applied Biosystems/Life Technologies′ guidelines. Results were normalized to β2-microglobulin expression and expressed as arbitrary units using one of the untreated samples as a calibrator as specified in the figure legend.

### 2.11. HIF-1α Enzyme-Linked Immunosorbent Assay (ELISA)

HIF-1α was measured using a commercial ELISA kit (R&D systems/ Biotechne, UK) following the manufacturers′ instructions. Endothelial cells were lysed in 80 μL lysis buffer (25 mmol/L Tris HCl pH 7.6, 0.1% SDS, 1% deoxycholate, 1% NP40, 0.5 mol/L EDTA, 40 mmol/L EGTA and protease inhibitors). Lysates were then centrifuged at 11,000× *g* for 15 min at 4 °C and the supernatant was collected. Protein concentrations were quantified using a BCA reagent kit (Pierce Biotechnology, Rockford, IL, USA) Results are expressed as pg/mg protein.

### 2.12. Statistical Analysis

All values were evaluated by one-way analysis of variance followed by Bonferroni′s multiple comparison tests (GraphPad Prism 7). Significant differences were assumed at *p* < 0.05.

## 3. Results and Discussion

### 3.1. Bioink Formulation and Assessment: 3D Printing 

All bioprinting inks, as shown in Table 1, were prepared by blending the optimized amount of either F127 or PLA/PEG mixed with the drugs. Viscosities of the different bioink compositions or drug loaded formulations were compared with those without the drugs. As can be seen from Table 1, the addition of the drug solutions to the actual ink formulations resulted in slightly lower viscosity. Blank formulations without the drugs showed quite high viscosity values of 74 and 120 Pa·s for F127 and PLA/PEG systems, respectively. Upon loading the drug into the formulations, the viscosity seemed to be reduced to 60 and 100 Pa·s for the F127 and PLA/PEG formulations, respectively. This could be attributed to the low-viscosity solution of the drug affecting the original viscosity of the blank polymeric pastes. Nonetheless, the slight observed reduction in the viscosity values did not significantly affect the actual printing process. None of the ink formulations were autoclaved prior to the actual printing, as in our previous screening study it had been found that autoclaving did not significantly alter the viscosity. Therefore, all scaffolds were fabricated by using the ink formulations as received without prior autoclaving. Moreover, all compositions showed shear thinning effects at increasing shear rates, enabling extrusion through nozzles, evidenced by the inks (Figure 1). 

All ink formulations exhibited satisfactory plotting behavior during the actual bioprinting process, as only optimized formulations were used for the purpose of this study. All optimized formulations as shown in Table 1 allowed plotting via the 3D printing process with high shape fidelity. Those ink formulations with drugs were extrudable at lower pressures compared to the blank, because the addition of the drug solutions slightly affected the printing fidelity. 

**Figure 2:**
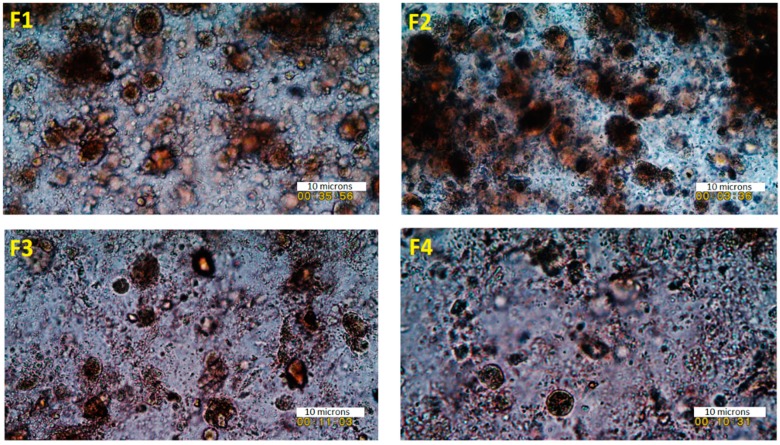
The texture and morphology of the semi-solid bioinks under an optical microscope of formulations F1 (F127:drug 4:1), F2 (PLA/PEG:drug 49:1), F3 (F127:drug 1:0), and F4 (PLA/PEG: drug 49:0) (scale 10 microns).

As expected, the F127, as well as PLA/PEG blend, was also able to generate scaffolds with excellent shape fidelity with moderate mechanical pressure in the system for extrusion. This is because of the texture and homogenous composition of the bioink formulations, where drug particles are miscible with the polymeric carrier (Figure 2 and Supplementay Materials Figure S1 and S2). The particles of the drug on the carrier polymeric matrices in the bioinks were homogeneously distributed throughout the matrices. The average size of the particles in the developed viscous ink formulations was below 10 microns, which is adequate for extrusion through a 22-gauge needle during the 3D printing process (Figure 2). The rationale of the optical microscopic images was to show the dispersion of the particles (of each of the components) in the ink formulations. As can be seen in Figure 2, all ink formulations showed sub-micron particles dispersed throughout the tested specimen. The overall findings from these images suggest that all particles were distributed and dispersed throughout the formulations.

**Figure 3:**
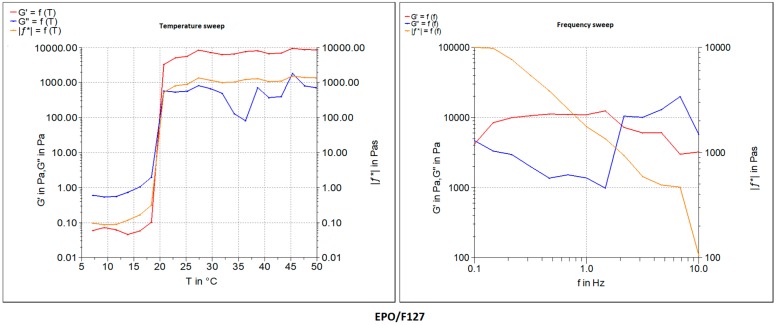
Rheology data of the bioink formulations represented by storage modulus (G′) and loss modulus (G′′) both in temperature (test run at 5–50 °C) and frequency sweeps (test run at 25 °C) for formulation F127/EPO 4:1.

Moreover, the pastes were found to have viscoelastic behavior and were tested at an amplitude in the viscoelastic region, determined by the temperature and frequency sweep tests. Figure 3 shows the oscillatory frequency and temperature sweep tests of the developed formulations with both F127 and PLA/PEG compositions in comparison with the blank polymeric bioink (F127 and PLA/PEG alone without the drugs EPO or DMOG). The storage modulus G′ indicates the elastic modulus, gelled component, while the loss modulus G′′ describes the viscous modulus, non-gelled component of the bioink formulations. The F127 bioink formulations showed a higher storage modulus compared to that of the of loss modus over a broad range of temperature and frequencies (as well as angular velocities). For F127 formulations, the G′ increased up to 10 KPa whereas the G′′ value decreased 10-fold. Interestingly, the storage modulus G′ showed a plateau kind of elastic behavior after it reached the highest value at above 25 °C attributed to the phase transition of the thermoresposnsive polymer. A further increase in the temperature did not have any impact on the increase on the G′ values of the F127 formulations. In contrast, a slightly different phenomenon was observed in the frequency sweep tests with the F127 formulations where the G′ seemed to have decreased with the increase in the frequency whereas G′′ showed an increased profile with the increase in the frequency during the test. Quite similar viscoelastic profiles were also observed in the PLA/PEG systems except for the plateau elastic points. At this crossover point, the bioinks seemed to have lost its viscous properties and behaved like an elastic solid, evidenced by the higher shear modulus. Plotting of all developed bioinks as shown in Table 1 was successfully performed without any stabilizing liquid by extrusion using a dosing needle with an inner diameter of ~640 μm. After the optimization of the printing process, all circular (10 mm diameter) constructs were successfully developed with high accuracy in dimensions. These scaffolds were suitable for cell culture with the potential for clinical applications (Figure 1).

### 3.2. Surface Morphology and Characterization of Scaffolds 

**Figure 4:**
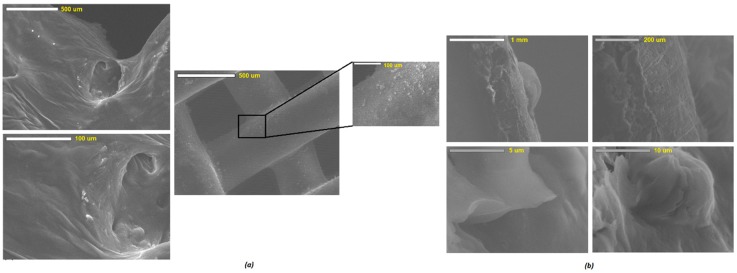
SEM images of (**a**) bioprinted, and (**b**) cross-section of PLA/PEG/EPO-based scaffolds.

SEM examined the surface morphology of the developed scaffolds. The results showed a relatively smooth surface for all scaffolds prepared. SEM analysis of both the F127 and PLA/PEG-based scaffolds revealed differences of the microstructure between the outer surface and the inner structure of the strands. Figure 4 shows a view of the observed surfaces which are smooth surfaces. The surface morphology was unaltered over the period of cell culture analysis. There are some small particles seemed to have been adsorbed onto the surface of the scaffolds matrices which could be attributed to the loose particles deposited during the or post-printing process. The lateral view of sliced scaffolds as shown in Figure 4a displayed uneven strand structures with rough areas. The rough surfaces appeared to be porous, and more micro-particles clumped together to form large agglomerates, while the smooth surfaces exhibited a dense and compact texture. A further analysis conducted via confocal microscopy revealed a homogenous particle distribution on the surface of the 3D printed scaffolds. The advanced analysis of the confocal microscopy was performed mainly in order to determine the distribution of the drug and overall homogeneity of the scaffolds. As can be seen in Figure 4b, most of the phases showed a similar set of textures due to the homogeneous distribution of the combined substances represented by the fluorescent dye coating of the scaffolds. It is expected that owing to the similarity of the deposition mechanism of the drug into the scaffolds during the actual printing, this homogeneous distribution would be analogous to the drugs. 

### 3.3. Mechanical and Thermal Analysis 

**Figure 5:**
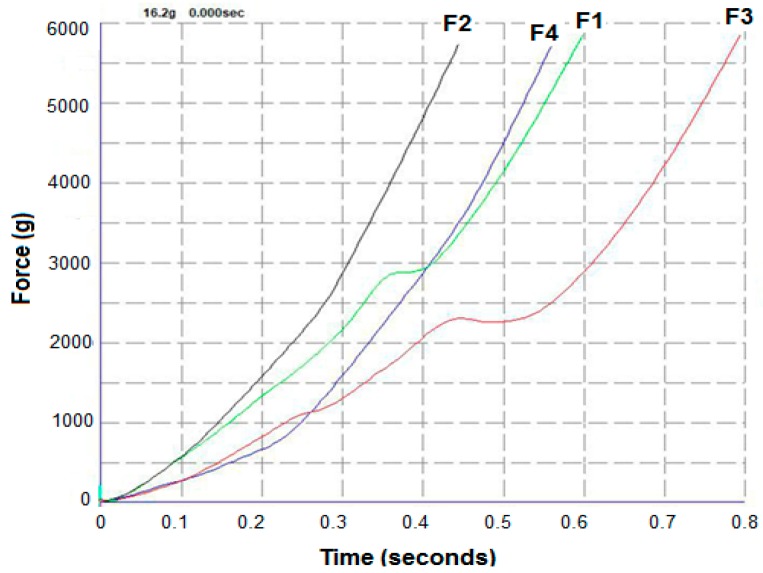
Force and travel time (as function of strain) profiles of the bioink formulations: F1 (F127:drug 4:1), F2 (PLA/PEG:drug 49:1), F3 (F127:drug 1:0), and F4 (PLA/PEG: drug 49:0) prior to the 3D printing applications (n = 3) of porous scaffolds.

The mechanical analysis revealed that all the developed formulations showed robust properties. As can be seen in Figure 5, the profiles of Force and % strain of all the ink formulations showed a strength of about 55–60 N for all of the bioinks. It appears that the addition of the drug solutions to the formulations did not have any negative impact on the force values. In contrast, the addition of drug solutions resulted in a significant decrease in the % strain values. The blank polymeric ink formulations showed a relatively high % strain between 80–110 whereas the actual drug loaded formulations showed only between 55%–60%. This could be attributed to the inclusion of low-viscosity aqueous solution in which the drug was dissolved in the actual formulation during the printing process. Interestingly, the presence of pluronic F127 in the formulations exhibited an additional peak force at about 30% strain when F127 was used alone or at 28% strain when used with drug solution. This could potentially be attributed to the thermoresonsive nature of the polymer, as F127 viscous solution has a phase transition temperature around the ambient (>25 °C). Therefore, during the texture analysis testing, F127 might have undergone a phase transition and had become more robust at 28%–30% strain. After this critical point, as expected the strain profiles were seen increasing throughout the rest of the testing period. Nonetheless, the reduction in the % stain did not have any significant effect on the printability and characterization of the resulting scaffolds. 

**Figure 6:**
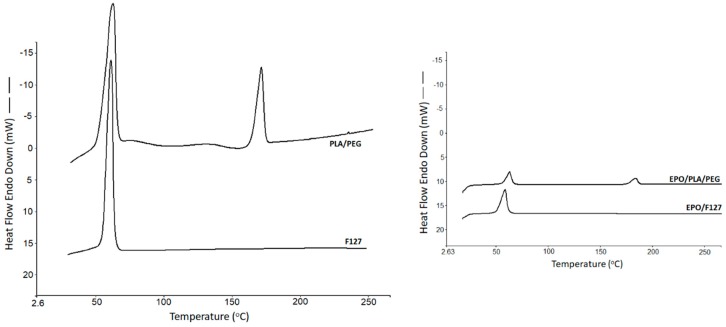
DSC thermal transitions of the blank polymeric formulations and the scaffolds.

The solid state of the drug loaded scaffolds as well as blank scaffolds were studied using a differential scanning calorimetry (DSC) analysis. The blank F127 exhibited a sharp thermal transition at 61.2 °C (∆*H* = 135.18 J/g), which is attributed to it melting, whereas the PLA/PEG blank system showed two endothermic transitions, one at 61.65 °C (∆*H* = 42.69 J/g) and 168.45 °C (∆*H* = 17.56 J/g) due to the melting of PEG and PLA, respectively (Figure 6). The presence of two distinct endotherms in the PLA/Peg system simply indicates the co-existence of two different crystalline phases coming from each of the polymers. The glass transition temperature of PLA was not visible, possibly owing to the enthalpy relaxation or overlapping with the melting endotherm of the low melting point PEG. For the nature of this study, no further investigation was made on this thermal event and enthalpy relaxation phenomenon. The scaffolds showed similar kinds of thermal events, where the peak intensity seemed to have been reduced. This could be attributed to the presence of the drug solution in the scaffolds. The drug solution exhibited some plasticization effects reflected by a slight reduction in the temperature and the intensity of the respective peaks at which the thermal event had occurred. As a result, the F127-based drug-loaded scaffold exhibited a melting endotherm at a slightly lower temperature at 60.78 °C with the heat of fusion value of 126.21 J/g. The PLA/PEG scaffolds showed two low intensity endothermal transitions at 63.89 °C (∆*H* = 41.38 J/g) and 169.16 °C (∆*H* = 25.12 J/g), respectively.

### 3.4. Drug Release and Its Biological Activity

**Figure 7:**
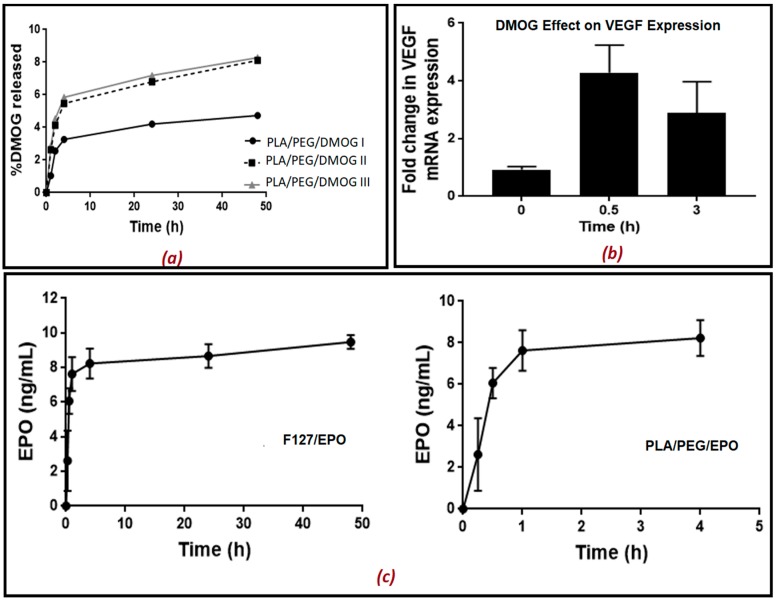
The release of HIF-1α inducer; (**a**) DMOG from 3D printed scaffolds in phosphate buffered saline solution (PBS) at room temperature. DMOG was used at a concentration of 30% in all the matrices prepared. (The biomaterial mixture contained PLA/PEG in the ratio 70:0, 60:10 and 50:20), (**b**) Fold change in VEGF gene expression in cell lysates after treating rat aortic endothelial cells with different samples of DMOG released at different time points (0, 0.5 and 3 h), and VEGF gene expression VEGF mRNA levels were measured as arbitrary units versus the untreated samples. (**c**) release of EPO from the 3D printed scaffolds. Data are the mean ± SEM of 6 samples. *P* < 0.01.

The in vitro release of the both EPO and DMOG was measured over 48 h. The concentration of DMOG was quantitatively measured indirectly by measuring HIF-1α released after adding aliquots containing the released drugs at different time intervals on rat aortic endothelial cells (RAECs) for 2 h. The concentration of EPO released was also quantitatively measured directly using an ELISA kit without adding on RAECs. The presence of PEG in the formulation helped trigger the release of the drugs from the scaffold matrices. To assess the effect of PEG in the formulations, three different kinds of PEG concentrations were used for the analysis of DMOG release from the scaffolds (Figure 7a,b). As can be seen in Figure 7a, the release was faster as PEG concentration increased, and PLA concentration decreased in the biomaterial matrix prepared. For the comparison purposes, when PLA alone was used, DMOG concentration reached 4% after 48 h which was as expected owing to the release retarding nature of the polylactide polymer. Interestingly, the presence of PEG in the formulations increased the release of the drug, to a certain extent, and approximately 8% DMOG release was observed when PEG concentration was 20% (*w*/*w*). This release pattern indicates that these scaffolds can potentially be used for biodegradable medical implants where a slow release for a prolonged period is desired. Similarly, the in vitro release of EPO from both the F127 and PLA/PEG matrices showed slow release of the drug over 48 h. As can be seen in Figure 7c, only about 9 ng/mL of EPO was released after 48 h in F127-based formulation whereas a little bit of faster release was observed for PLA/PEG formulation. As can be seen in Figure 7c, about 8 ng/mL of EPO was released in only 2 h. Nonetheless, both PLA/PEG and F127 formulations showed a similar kind of release patterns. Though the presence of PEG in the formulations would be expected to help propagate the release, this has not been evident in this study. The slower release from the F127 formulations could be attributed to the possible strong entrapment of the drug within the scaffolds. For the full analysis, a longer period of study needs to be fully executed. However, since the aim of this study was to assess the suitability of the emerging bioprinting and its potential applications in endothelial cell repair no further formulation optimization was undertaken. 

Moreover, a further investigation was conducted to study if DMOG released from the scaffolds was enough to cause a significant increase in HIF-1α levels when incubated with RAECs for 2 h and resulted in transcriptional activation of HIF-1α target genes (VEGF). The results presented in Figure 7b indicated that 30 min was the optimum time for the release of DMOG from the scaffold causing an increase in the expression of the VEGF gene by 3–4 fold. This was like the effect of standard DMOG solution added in a concentration of 100 µM (Supplementary Materials Figure S3). After 3 h, the DMOG released still caused a significant increase in VEGF gene expression compared to untreated cells. Moreover, it was reported elsewhere that the optimum bioactive concentration of DMOG is 100 uM [22]. In conclusion, it can be claimed that our optimized bioprinted scaffolds showed controlled release of both EPO and DMOG when used individually. As EPO′s erythropoietic effects may increase the risk of thrombosis when EPO is administered to non-anaemic patients alone, a synergistic release of both EPO and DMOG from the same scaffold would provide an excellent alternative beyond the current state of the art. It has been reported that the hypoxia enhances the reparative response of ECs to EPO and its analogues which would likely be mediated by hypoxia inducible factor (HIF)-1α such as DMOG [24]. It can therefore be claimed that the endothelial repair would occur using a combination of EPO (the prototypic TPC) and DMOG (a HIF-1α inducer) without promoting neo-intimal growth. The foregoing will be explored in follow-on studies.

## 4. Conclusions

The optimized bioink formulations represent an intriguing alternative for 3D bioprinting materials when loaded with drugs. In this paper, we successfully exploited the use of emerging 3D bioprinting techniques for the development and optimization of novel bioinks of biomaterials like F127 and PLA. As a result, a novel composite bioink from PLA/PEG has been synthesized and characterized. All developed bioinks exhibited excellent printability and bioink properties indicated by printing/plotting fidelity. Moreover, the 3D printed constructs showed homogenous distribution of the drugs in the scaffolds without compromising the mechanical and thermal properties of the scaffolds. Further experiments are ongoing to confirm the final interaction between the components used in the formulations and further optimise it for the higher release of the drugs from the scaffolds. Moreover, the bio-functional benefits of the materials used in the formulations were evidenced by the sustained release of the model drugs and VEGF. The current studies demonstrate the exciting potential of our developed semi-solid formulations as a robust, and reliable bioink for 3D printing in biomedical applications.

## Figures and Tables

**Table 1:** Formulation compositions of printing inks containing PEO and DMOG (*w*/*w* ratio).

Formulations	Pluronic F127	PLA/PEG Mixture	EPO (2 µg/mL)/DMOG	Peak Positive Force (N)	Viscosity (cP)	Injectability
1	4	0	1	56.604	7.4 × 10^3^	Pass
2	0	49	1	55.253	10.0 × 10^3^	Pass
3	1	0	0	56.585	6.0 × 10^3^	Pass
4	0	49	0	55.038	12.0 × 10^3^	Pass

## Data Availability

The raw/processed data required to reproduce these findings cannot be shared at this time as the data also forms part of an ongoing study.

## Supplementary Materials

The following are available online at https://www.mdpi.com/2073-4360/11/12/1924/s1, Figure S1: Syringability tests of prepared bioinks containing (a,b) PLA/PEG; Figure S2: Texture of the prepared homogenous bioinks in high magnification (F127/drug); Figure S3: Fold change in VEGF gene expression in cell lysates after treating rat aortic endothelial cells with standard DMOG solution added in a concentration of 100 μM.

## Fundings

This research received no external funding.
