# Supplementary material for: 3D Bioprinting of Novel Biocompatible Scaffolds for Endothelial Cell Repair
Source: Polymers (Basel). 2019 Nov 22;11(12):1924. doi: 10.3390/polym11121924 (PMC6960937; doi:10.3390/polym11121924)
Supplement: Supplementary file 1 [file polymers-11-01924-s001.pdf]

polymers

MDPI

3D bioprinting of novel biocompatible scaffolds for endothelial cell repair

Yan Wu[1], Lamia Heikal[2,3], Gordon Ferns[2], Pietro Ghezzi[2], Ali Nokhodchi[1,4*], Mohammed Maniruzzaman[5] and *

[1] Department of Pharmacy (Chemistry), School of Life Sciences, University of Sussex, Falmer, Brighton, BN1 9QJ
[2] Brighton and Sussex Medical School, Falmer, Brighton, BN1 9RH
[3] Faculty of Pharmacy, Department of Pharmaceutics. Alexandria University, Egypt
[4] Drug Applied Research Center and Faculty of Pharmacy, Tabriz University of Medical Sciences, Iran
[5] Pharmaceutical Engineering and 3D Printing (PharmE3D) Lab, Division of Molecular Pharmaceutics and Drug Delivery, College of Pharmacy, University of Texas at Austin, 2409 University Avenue, A1920, Austin, Texas, 78712, USA
* **Correspondence:** M Maniruzzaman: M.Maniruzzaman@austin.utexas.edu ;M.Maniruzzaman12@gmail.com, and A. Nokhodchi: A.Nokhodchi@sussex.ac.uk

polymers
MDPI

Supplementary Data:

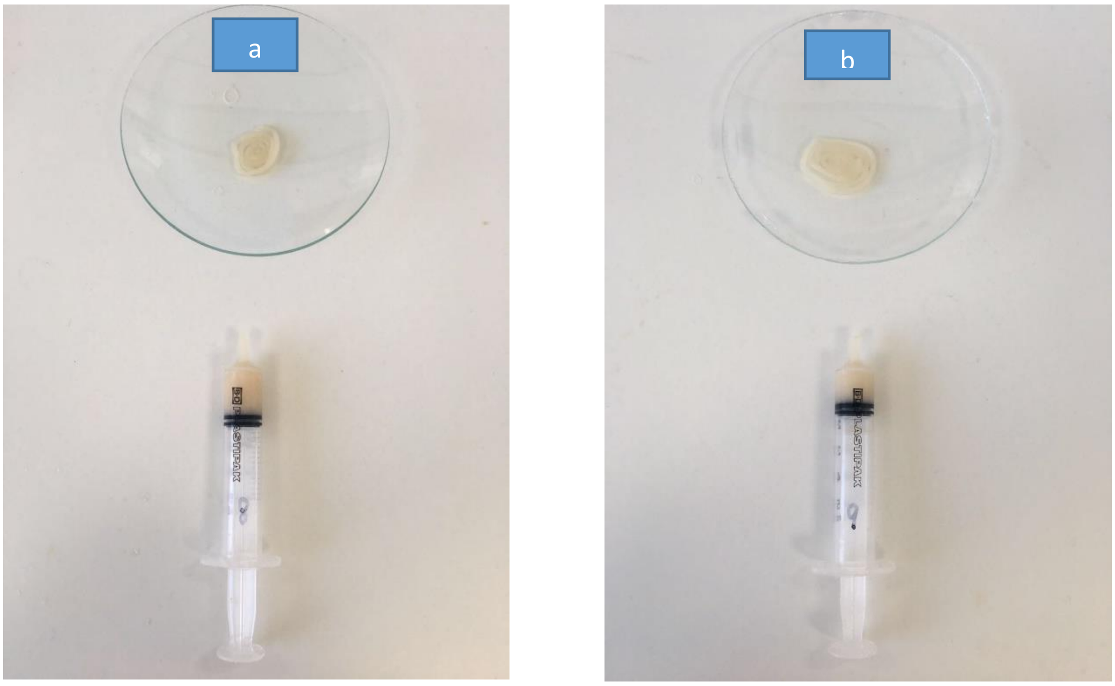

Supp. Fig. 1: Syringability tests of prepared bioinks containing (a-b) PLA/PEG.

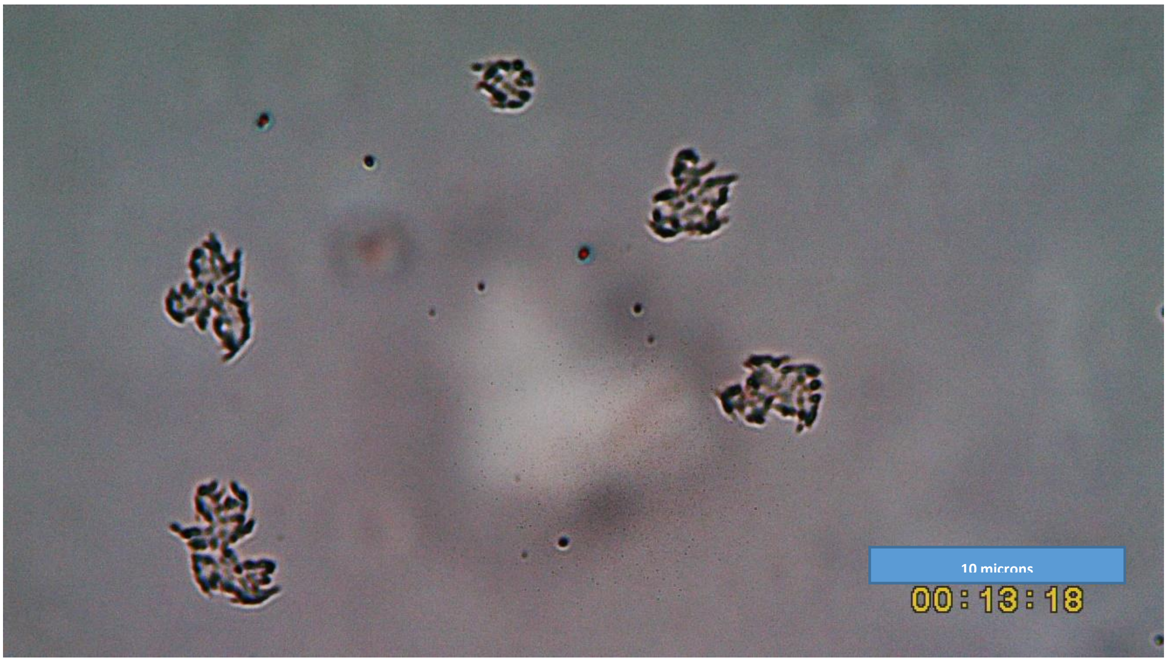

Supp. Fig. 2: Texture of the prepared homogenous bioinks in high magnification (F127/drug).

polymers

MDPI

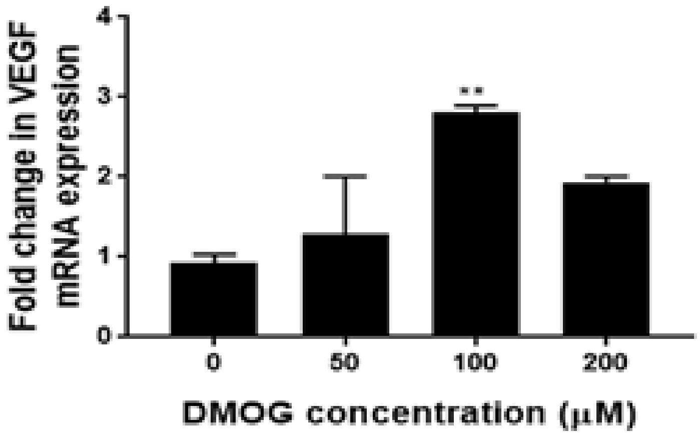

Supp. Fig. 3: Fold change in VEGF gene expression in cell lysates after treating rat aortic endothelial cells with standard DMOG solution added in a concentration of 100 µM.
